# Associations between Cardiovascular Outcomes and Rheumatoid Arthritis: A Nationwide Population-Based Cohort Study

**DOI:** 10.3390/jcm11226812

**Published:** 2022-11-17

**Authors:** Seonyoung Kang, Kyungdo Han, Jin-Hyung Jung, Yeonghee Eun, In Young Kim, Jiwon Hwang, Eun-Mi Koh, Seulkee Lee, Hoon-Suk Cha, Hyungjin Kim, Jaejoon Lee

**Affiliations:** 1 Department of Medicine, Samsung Medical Center, Sungkyunkwan University School of Medicine, Seoul 06351, Republic of Korea; 2 Department of Statistics and Actuarial Science, Soongsil University, Seoul 06978, Republic of Korea; 3 Department of Medical Statistics, College of Medicine, Catholic University of Korea, Seoul 06591, Republic of Korea; 4 Division of Rheumatology, Department of Internal Medicine, Kangbuk Samsung Hospital, Sungkyunkwan University School of Medicine, Seoul 03181, Republic of Korea; 5 Department of Medicine, National Police Hospital, Seoul 05715, Republic of Korea; 6 Division of Rheumatology, Department of Internal Medicine, Sungkyunkwan University Samsung Changwon Hospital, Changwon 51353, Republic of Korea; 7 Korean Health Insurance Review and Assessment Service, Seoul 06653, Republic of Korea; 8 Department of Medical Humanities, Samsung Medical Center, Sungkyunkwan University School of Medicine, Seoul 06351, Republic of Korea

**Keywords:** rheumatoid arthritis, cardiovascular disease

## Abstract

Despite a growing burden posed by cardiovascular disease (CVD) in rheumatoid arthritis (RA) patients, large-scale studies on the association between the characteristics of RA patients and CVD risks and studies adjusted for various confounding factors are lacking. In this large-scale nationwide cohort study, we aimed to investigate the association between CVD risk and RA and factors that may increase CVD risk using a dataset provided by the Korean National Health Insurance Service (NHIS). We enrolled 136,469 patients with RA who participated in national health examinations within two years of RA diagnosis between 2010 and 2017 and non-RA controls matched by age and sex (*n* = 682,345). The outcome was the occurrence of myocardial infarction (MI) or stroke. MI was defined as one hospitalization or two outpatient visits with ICD-10-CM codes I21 or I22. Stroke was defined as one hospitalization with ICD-10-CM codes I63 or I64 and a claim for brain imaging (CT or MRI). The Cox proportional hazard model and Kaplan–Meier curve were used for analysis. The mean follow-up duration was 4.7 years, and the incidence rate of CVD was higher in the RA group than the control group (MI: 3.20 vs. 2.08; stroke: 2.84 vs. 2.33 per 1000 person-years). The risk of MI and stroke was about 50% and 20% higher, respectively, in RA patients. The association between RA and CVD was prominent in females after adjusting for confounding variables. The association between RA and risk of MI was significant in individuals without DM. Therefore, appropriate screening for CVD is important in all RA patients including females and younger patients.

## 1. Introduction

Rheumatoid arthritis (RA) is a systemic inflammatory disease with high mortality and morbidity rates that are mainly attributable to cardiovascular disease (CVD) [1]. RA is considered an independent CVD risk factor by the European Society of Cardiology guidelines, and the extent of increased CVD risk in patients with RA is up to two-fold higher than that in the general population, nearly equivalent to that of type 2 diabetes mellitus (DM) [2,3]. The increased CVD risk is observed in patients with early RA and even prior to diagnosis of RA (subclinical RA), which is not fully explained by differences in traditional CVD risk factors or RA-related risk factors at diagnosis [4]. 

Several epidemiologic studies have investigated the association between CVD risk and RA. Previous cohort studies conducted in western countries reported that RA was linked to a higher risk of CVD, with a 1.4- to 3.7-fold increased risk for myocardial infarction (MI) and 1.3- to 2.7-fold increased risk for stroke [5,6,7,8,9,10,11,12,13,14]. However, research on modifiers associated with increased CVD in RA has yielded conflicting results. Some studies reported that younger age was associated with increased CVD in RA subjects [5,13]. A Korean nationwide cohort study of 2765 RA patients showed that increased MI risk in RA was associated with non-diabetes, and higher risk of stroke in RA was associated with female sex, non-diabetes, and non-dyslipidemia [15,16]. In a large UK case–control study of 6591 RA patients, current smoking, body mass index (BMI), and diabetes were associated with higher CV risk in RA patients [4]. In a study of postmenopausal women, joint pain and WBC count were significantly associated with CVD [17]. Another study reported that anti-CCP positivity was associated with increased ischemic heart disease and fatal CVD [18]; however, a different study showed no association between coronary atherosclerosis and anti-CCP antibody positivity [19]. 

In contrast to data showing increased risk of MI among RA patients [5,6,9,10], studies on the association of RA with stroke have found conflicting results [7,11,13,14,20,21,22,23]. Some studies reported increased risk of stroke in RA [14,20,23], but others failed to demonstrate a difference [7,11,13,21,22]. In a Nurses’ Health Study of 114,342 women, RA subjects had increased risk of MI but a similar risk of ischemic stroke compared with subjects without RA [14]. Similarly, Turesson et al. reported a larger risk of incident stroke (combined ischemic and hemorrhagic stroke) in RA subjects, but the difference was not statistically significant [23]. In a Swedish rheumatology cohort, the rate of ischemic stroke was approximately 30% higher in RA patients than in the general population (aHR 1.29, 95% CI 1.18–1.41) [7]. 

To date, accumulating evidence has revealed that incident CV risk is higher in RA patients compared with the general population. However, the extent of the increase in CV risk differs among studies, and which characteristics of RA patients are mainly associated with increased CV risk remains unclear. Among studies to date, the largest sample size was 114,342 subjects, but the study only included female subjects and used self-reported questionnaires to define RA, CVD, and other covariates [14]. Another large-scale study of 160,000 postmenopausal women mainly reported the association between anti-CCP antibody and CVD [17]. Unfortunately, many previous studies on association between RA and CVD were conducted on non-Asian groups. Only three epidemiological studies have investigated this issue in a South Korean population, but the data were evaluated in a cross-sectional manner or in models that were not fully adjusted with a small number of subjects. Therefore, we aimed to investigate associations between CV outcomes and RA and explore the characteristics of RA patients that are associated with higher CV risk in a large, nationwide, longitudinal population-based cohort of South Korean patients after adjusting for various confounding factors and using validated definitions of variables. 

## 2. Materials and Methods

### 2.1. Data Source

The study used a dataset provided by the National Health Insurance Service (NHIS) in Republic of Korea. The NHIS is an insurance provider managed by the government that covers 97% of the population, approximately 50 million people. The NHIS dataset includes information on socioeconomic, demographic variables, health care utilization, health examination findings, disease diagnosis under the ICD-10 Clinical Modification (ICD-10-CM), and medical treatment and procedure. NHIS offers a standardized health examination at least every two years [24]. The NHIS database linking claims data and health examination data, which include a variety of medical and health information, has been widely used in various epidemiologic studies, including retrospective cohort studies, and also used to identify risk factors for diseases [25,26].

### 2.2. Study Population

We included adults over the age of 19 with a diagnosis of RA between 1 January 2010 and 31 December 2017 (*n* = 286,906). Among them, patients who underwent a health examination within two years before the first diagnosis of RA and were matched by age and sex to individuals without RA (using a 1:5 ratio) were included. RA was defined as at least one claim under ICD-10-CM code M05 or M06 and at least one claim for prescription of any DMARD. The validity of these algorithms was evaluated in previous studies, which found a positive predictive value > 0.82 [27,28].

We excluded patients who had an alternative diagnosis (psoriatic arthritis, ankylosing spondylitis, or other spondyloarthropathy, systemic lupus erythematosus, juvenile idiopathic arthritis), with any missing variables of interest. We also excluded patients with a diagnosis of MI or stroke before enrollment to increase the accuracy of primary endpoint diagnosis. A history of MI or stroke was defined using ICD-10-CM codes (I21, 22 for MI, I63, 64 for stroke) regardless of admission. This study protocol followed the Declaration of Helsinki and was approved by the Institutional Review Board of Samsung Medical Center (IRB File No. SMC 2021-12-083). This study did not require informed consent because no identifiable patient information was available to researchers.

### 2.3. Data Collection

The date of diagnosis of RA was defined as the index date. The index date of controls was defined as the RA diagnosis date of their matched counterpart. Baseline comorbidities such as hypertension, diabetes, and hyperlipidemia were collected based on the combination of health examination results and ICD-10-CM diagnosis and prescription information. Hypertension was defined as systolic/diastolic BP ≥ 140/90 mmHg, ICD code I10–13 or I15, and claims for antihypertensive medication prescription. Diabetes was defined as at least one claim of the E11–14 code and at least one antidiabetic medication prescription or a fasting plasma glucose level ≥ 126 mg/dL. Hyperlipidemia was defined as E78 code and at least one prescription claim of lipid-lowering medication or serum total cholesterol level ≥240 mg/dL. Chronic kidney disease was defined as estimated glomerular filtration rate <60 mL/min/1.73 m^2^. Through the health examination, body weight, waist circumference, and blood pressure were measured, and laboratory test items such as fasting glucose, total cholesterol, and creatinine were included. From the self-reported questionnaire, demographic and lifestyle data were collected, including data of smoking, alcohol, and regular exercise. Smoking status was divided as smoker (current smokers) and nonsmoker (never and ex-smokers). Subjects with an intake ≥30 g of alcohol per day were classified as heavy alcohol drinkers. Regular exercise was defined as moderate physical activity for ≥30 min at least five times per week or vigorous physical activity for ≥20 min at least three times per week. Income level was classified into the lowest 20% of income. These definitions were based on previous studies [24,29].

### 2.4. Study Outcomes and Follow up

The endpoint of the study was newly diagnosed CVD. MI was defined as an ICD-10-CM code of I21 or I22 with one hospitalization. The validity of this definition was evaluated in prior studies with a positive predictive value (PPV) of 92% [30]. Stroke was defined as a I63 or I64 code with one hospitalization and a claim for brain imaging (CT or MRI). This algorithm was widely adopted in previous studies using claims databases with a PPV of 92.2% [30]. The study participants were followed from the index date until the occurrence outcome or study end date (31 December 2019), whichever came first.

### 2.5. Statistical Analysis

Data were statistically analyzed using SAS version 9.4 (SAS Institute Inc., Cary, NC, USA). The baseline characteristics of the study population are presented as mean ± standard deviation (SD) for continuous variables, and categorical variables are expressed as numbers and percentages. The incidence rates of MI and stroke were calculated by dividing the number of incident events by the total follow-up period (1000 person-years). Hazard ratios (HRs) and 95% confidence intervals (CIs) for the risk of incident CVD were calculated by the Cox proportional hazard model. In multivariable analysis, the following confounders were adjusted: age, sex, smoking, alcohol drinking, regular exercise, obesity, DM, HTN, hyperlipidemia, CKD, and income. In addition to multivariable analysis, propensity score matching (PSM) analysis was adopted to adjust for disparities in baseline characteristics between the RA and non-RA cohorts. Selection bias can be reduced using PSM [31]. Kaplan–Meier survival curve analysis was used to analyze the cumulative incidence of CVD according to RA status. To investigate the effects of potential modifiers on the association between RA and CVD, subgroup analysis was performed according to various factors (age, sex, smoking, alcohol drinking, physical activity, obesity, DM, HTN, hyperlipidemia, and CKD). These factors were selected based on clinical experience, previous literature, and evidence that demonstrated an association with CVD [5,13,32,33,34,35]. A *p* value less than 0.05 was considered statistically significant.

### 2.6. Data Availability Statement

The datasets generated during the current study are available from the corresponding author upon reasonable request.

## 3. Results

### 3.1. Study Population

**Figure 1:**
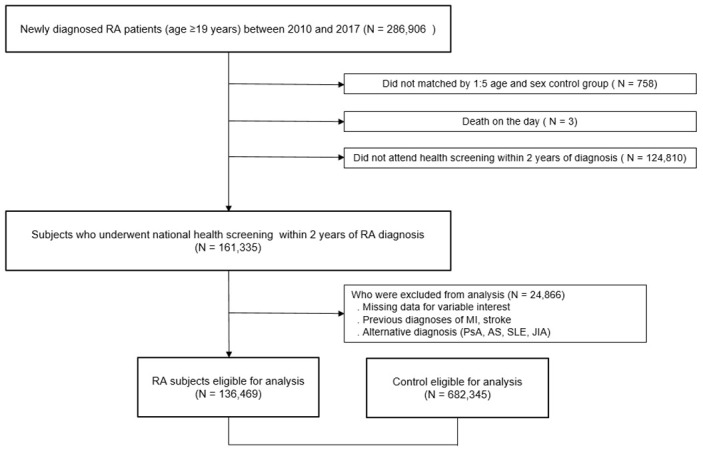
Flowchart of the study population. RA; rheumatoid arthritis.

Of the 286,906 subjects with newly diagnosed RA (≥19 years of age) in the dataset, there were 286,148 RA subjects with age- and sex-matched counterparts, and 161,335 RA subjects with examined health check-up 2 years before the index date. After applying the exclusion criteria, 136,469 RA subjects were included in the analysis. A total of 683,345 age- and sex-matched individuals was analyzed as controls. The flowchart of the study population selection is shown in Figure 1.

### 3.2. Baseline Characteristics

Table 1 shows the baseline characteristics of the study population (including 136,469 individuals with RA and 682,345 individuals without RA). Compared with individuals without RA, individuals with RA had a higher rate of some traditional CV risk factors, including low physical inactivity, diabetes, and hypertension. However, the proportions of smokers and obesity were higher in the controls.

### 3.3. Clinical Outcomes in the Original Cohort

**Figure 2:**
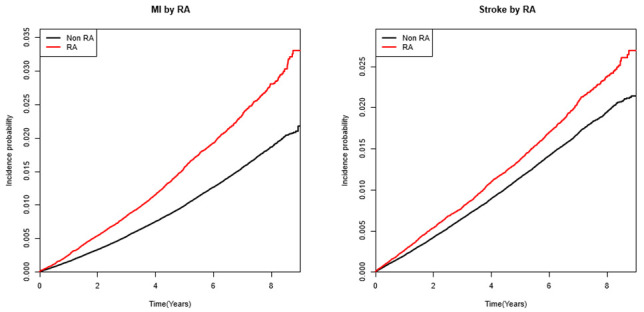
Cumulative incidence curve of CV events in patients with RA and non-RA control individuals.

Table 2 presents the cumulative incidences (risk) per 1000 persons and HRs for the study outcomes, and cumulative incidence curves are provided in Figure 2. The mean follow-up duration for MI was 4.75 ± 2.22 and 4.72 ± 2.23 years in the RA group and control group, respectively. For stroke, the mean follow-up duration was 4.74 ± 2.23 and 4.71 ± 2.23 years, respectively. In the RA cohort, during follow-up, 2061 subjects were newly diagnosed with MI, and the incidence rate of MI was 3.20 per 1000 person-years (control incident rate 2.08). For stroke, 1830 RA subjects were newly diagnosed, and the incidence rate was 2.84 per 1000 person-years (control incident rate 2.33). The hazard ratios of MI and stroke in RA subjects were significantly higher than those in matched controls (aHR in MI 1.54, 95% CI 1.46–1.6; aHR in stroke 1.23, 95% CI 1.16–1.28). After adjusting for confounders including traditional risk factors, the HRs for the endpoints were not substantially attenuated.

### 3.4. Clinical Outcomes in the Propensity Score-Matched Cohort

In the original cohort, baseline characteristics were significantly different between RA and non-RA groups (Table 1). Thus, to balance the baseline covariates of both groups, we applied PSM. After 1:1 PSM, 272,936 subjects were included, with 136,468 subjects in each group and each measured baseline covariate was well balanced between groups (Supplementary Table S3). In the PS-matched cohort, the incidence rate of MI was 3.20 per 1000 person-years (control incident rate 2.10). For stroke, the incidence rate was 2.85 per 1000 person-years (control incident rate 2.33). RA was independently associated with increased risk of MI and stroke with a propensity score HR of 1.53 (CI 1.43–1.64), 1.22 (CI 1.14–1.31), respectively (Supplementary Table S4). Therefore, the clinical outcomes in the PSM model were almost identical to the results in the adjusted model of the original cohort.

### 3.5. Stratified Analysis

Stratified analysis on the association of RA with the risk of stroke and MI was performed based on various factors and the results are given in Supplementary Tables S1 and S2. The effect of RA on MI and stroke was greater in the younger group (MI: aHR 2.99 in <40 years, 1.52 in 40–64 years, 1.51 in ≥65 years, *p* for interaction < 0.0001; stroke: aHR 2.35 in <40 years, 1.22 in 40–64 years, 1.21 in ≥65 years, *p* for interaction = 0.0100). Similarly, we observed a significant association between MI and RA in females. The association of stroke with RA was pronounced in females (MI: aHR 1.42 in males, 1.60 in females, *p* for interaction = 0.0293; stroke: aHR 1.14 in males, 1.27 in females, *p* for interaction = 0.037). The association between RA and risk of MI was significant in individuals without DM (aHR 1.61 in DM, 1.30 in non-DM, *p* for interaction = 0.0005).

## 4. Discussion

In this large-scale study, RA was associated with increased risk of MI and stroke after adjusting for various confounders. Compared with subjects without RA, subjects with RA had 54% and 23% increases in the risk of MI and stroke, respectively. The results were similar after applying for PSM. The current results confirm those obtained in previous studies. In a 2012 meta-analysis, there was a 48% increased risk of incident CVD in RA subjects (pooled RR 1.48, 95% CI 1.36–1.62); the increased risks of MI and stroke were 68% (pooled RR 1.68, 95% CI 1.40–2.39) and 41% (pooled RR 1.41, 95% CI 1.14–1.74), respectively [1]. In a British Columbian cohort study of 24,385 RA patients matched with 242,976 non-RA patients, RA patients had an approximately doubled risk for MI and stroke in both sexes compared with age-matched counterparts [13]. The extent of increased risk was slightly higher than in our results. Unlike in our study, which applied previous CVD identified by ICD code as an exclusion criterion, the previous study had limited information on prior CVD and thus it is difficult to accept the outcomes as incident CVD. Thus, the rate of outcomes may have been overestimated. The first Asian cohort study performed in Taiwan found a 38% increased risk of MI in RA patients compared with non-RA subjects. In the study, the risk of MI in Taiwan was slightly lower than in western countries, which was interpreted as due to racial differences and long-term free use of DMARDs in Taiwan. Although that study was the first cohort study targeting Asians, it was conducted with a relatively small number of subjects and a short follow-up period (approximately 30,000 RA patients, 193,987 follow-up person-years), and risk factors such as smoking, obesity, and physical activity were not adjusted for [5]. In a Korean cross-sectional study of 238 RA subjects, RA was associated with an approximately two-fold higher prevalence of coronary artery disease (CAD; OR 2.97, 95% CI 1.15–7.68, *p* = 0.02). However, they defined RA and CVD according to self-reported questionnaires, in contrast to our study that used validated criteria [8]. Two recent Republic of Korea cohort studies of RA subjects showed similar results to ours, but with slightly different extent of increased risk (aHR for ischemic stroke 1.4, 95% CI 1.07–1.82; aHR for MI 4.21, 95% CI 2.86–6.19) [15,16]. The cohort protocols were similar to those of our study, but our study has several additional strengths. First, it included approximately five times more subjects and adjusted for various risk factors including obesity, CKD, smoking, alcohol, regular exercise, and income, which were not adjusted for in the previous study. Moreover, our study applied PSM to balance the distribution of baseline characteristics, resulting in more reliable outcomes. The previous Republic of Korea study only included seropositive RA patients, but we also included seronegative RA patients in our cohort. Regardless of the RF/ACPA positivity, chronic inflammation plays an important role in promoting accelerated atherogenesis. Atherosclerosis is a chronic inflammatory disease that shares similarities in pathogenesis and some genetic factors with RA [36]. In a 2017 meta-analysis, MI risk consistently increased across five major types of arthritis not fully explained by the prevalence of traditional risk factors, suggesting inflammation may be more important than the etiology of arthritis (RR 1.69, 95% CI 1.50–1.90 in RA; RR 1.41, CI 1.17–1.69 in PsA; RR 1.24, CI 0.93–1.65 in AS; RR 1.47, CI 1.17–1.69 in gout; RR 1.31, CI 1.01–1.71 in OA) [37].

The origin of excess risk and extent of the contribution of risk factors in RA cohorts have been major questions over the past decades [38]. The increased risk of CVD is not simply explained by a higher prevalence of classical risk factors [39]. This phenomenon is thought to be the combined effect of traditional CVD risk factors and multifactorial effects including systemic inflammation and autoimmune process in RA [40]. In an international cohort of 5638 RA patients in 2018, 30% of the increased CVD risk was explained by RA characteristics of DAS 28 level and RF/ACAP positivity, 40% was attributed to traditional risk factors, and the cause of the remaining 30% was unknown [41].

Systemic inflammation has been recognized as a central mechanism in the development of CVD, and this inflammatory response modulates other CVD risk factors, resulting in a special association between CV risk and RA compared with the general population [36]. Pro-inflammatory cytokines implicated in RA, such as tumor necrosis factor alpha (TNF-a) and interleukin-6 (IL-6), promote atherosclerotic processes by directly damaging the endothelium of vascular tissue through the modulation of classic risk factors and by interfering with the vascular repair system. Both innate and adaptive responses in inflammation influence the onset, progression, and destabilization of atherosclerosis [39,42,43]. In a clinical study, following Infliximab infusion, the percentage of cases with transient improvement of endothelial function (endothelial-dependent vasodilation) increased, suggesting that long-term TNF blockade reduces the high incidence of CVD complication in RA [44]. This mechanism is in line with previous studies on the relationship between disease severity and incidence of CVD [45,46,47,48,49]. A large body of evidence has shown that well-controlled inflammation is positively correlated with decreased CVD risk, and vice versa. In a cohort study in Minnesota, USA, CV mortality in RA was shown to improve over the past 20 years (2.7% in RA patients diagnosed 2000–2007, 7.1% in RA patients diagnosed 1990–1999), likely due to the more effective anti-rheumatic therapies such as various DMARDs and biologics, which contributed to lower disease activity by dampening the inflammation associated with lower cardiovascular risk [50]. In an early RA cohort in Nijmegen, low disease activity (DAS28 ≤ 3.2) was significantly associated with a reduced risk of CVD compared with DAS28 > 3.2. (HR 0.65, 95% CI 0.43–0.99) [51].

Some studies failed to demonstrate increased stroke risk in RA, but our study confirmed that the risk of ischemic stroke was higher in RA. This might be explained by the difference in stroke definition used in studies. Despite a discrepant risk factor profile and different pathogenesis between ischemic stroke and hemorrhagic stroke, some studies defined stroke using composite outcomes that included both ischemic and hemorrhagic stroke [20,23]. Considering the difference between ischemic and hemorrhagic stroke, it is necessary to classify stroke type and evaluate the risk of stroke among these two separate entities, rather than using a composite outcome. The present study evaluated the risk of stroke in RA subjects by limiting the definition to ischemic stroke.

In the subgroup analysis, there were significant interactions with age in the association between RA and CVD outcomes. The associations of RA with MI and stroke were stronger in the younger age group. The HRs of both events were highest in younger individuals with RA. However, as expected, absolute events increased with age. This suggests that RA might also be an important risk factor of major CVD, especially in younger individuals. The findings were similar to those obtained in a previous meta-analysis. The results of the meta-analysis revealed that, compared with the general population, the relative risks for CVD events in RA subjects are highest for younger patients, whereas older RA patients had a similar relative risk to age-matched controls (RR 2.59, 95% CI 1.77–3.79 in youngest patients; RR 1.27, 95% CI 1.16–1.38 in older patients) [52]. A Nijmegen 10-year follow-up cohort study of 836 RA patients reported that, when using current calculators for prediction of CVD risk (EULAR-modified SCORE), there was a discrepancy between predicted and observed CV risk in only younger RA subjects (5.3% predicted vs. 8.0% observed, in patients <55 years, *p* < 0.003). This suggests that CV risk under current risk algorithms developed for the general populations is especially underestimated in younger RA patients [53]. A British Columbian cohort study of 24,385 RA patients reported that the rate ratio of CV events was highest in younger age subgroups of RA patients (18–49 years old) in both sexes compared with their age-matched counterparts [13].

These risk patterns suggest several possible explanations. This finding highlights the concept of competing risk: as the population ages, relatively rare risk factors (such as RA) for CVD have less of an impact than other common risk factors. Hence, the rate ratio attributable to RA seems smaller with increasing age [13]. Another possibility is that unique factors in young RA patients such as smoking status, daily stress level, eating habits (e.g.,: red meat consumption) may be associated with a higher incidence of CVD than counterparts without RA [54]. Smoking, a traditional CV risk factor, has been traditionally associated with the development of seropositive RA and early onset RA [33,55]. In a cross-sectional study of 707 RA patients, high intake of red meat, which is positively related to incident CVD, was associated with early onset RA, especially in smokers or overweight patients [56,57].

In the current study, there were significant interactions with sex in the association between RA and major CVD events. The increased risk of MI and stroke associated with RA was prominent in women. This result is not unique. In previous studies, CVD has been recognized as a more common disease in women than men [58]. One possible explanation is that pregnancy-related disease such as gestational hypertension and DM as well as endocrine disorder (such as polycystic ovary syndrome and early menopause) that are more common in childbearing women may accelerate the onset of CVD [32]. In a cross-sectional study, high density lipoprotein (HDL) subfractions such as HDL3-chol and HDL2-chol, which have a protective effect against CVD, were reduced in female RA patients and not in male RA patients [59]. There have been several reports of an association among RA, early menopause, and CVD, suggesting that females with RA may have an additional adverse sex hormonal effect on RA development, disease severity, and CVD risk [60,61,62].

Some interesting results arose from the subgroup analyses. The incident rate of MI was higher in individuals with DM, both in the RA group and control group. However, the association of RA and MI was significantly higher in non-DM individuals than in DM individuals. A possible explanation is that the direct contribution of systemic inflammation attributable to RA may be higher in non-DM patients than those with DM. In other words, the role of diabetes related to MI may be very potent, and the relative contribution of RA is smaller among DM patients. Therefore, a preventative strategy of MI in RA patients should be emphasized regardless of DM.

Our study has a few limitations. First, PSM was applied and possible confounders were included in the multivariable model, but the possibility of unmeasured residual confounding factors cannot be excluded, and family history of CVD was not included. Moreover, additional statistical analysis such as nested case–control or risk set sampling was impossible in this study because of the characteristics of the NHIS database. Second, given the retrospective observational nature of the study, our findings cannot be used to prove a causal relationship. Third, we could not consider the severity of RA related to inflammation status because we did not have information on inflammatory markers such as C-reactive protein or disease activity score such as DAS-28, which are not collected in NIHS data. We also did not analyze the use of arthritis medications. Arthritis drugs are associated with CVD risk because they not only improve inflammation, but can also have adverse effects on CVD. Therefore, arthritis medication analysis would help elucidate CVD patterns and prevention in RA patients. Future research is warranted on the drug effects related to CVD. However, our study is meaningful because it identified the characteristics that affect the development of CVD in RA patients using a large population-based cohort that is representative of the demographic characteristics of the South Korea population. It also adjusted for various confounders and used a validated algorithm to define variables. We adopted PSM to minimize selection bias, which was not used in previous cohort studies. Data completeness without missing participants or variables of interest during a long-term follow-up period further strengthened this research, and stratified analysis yielded several interesting findings.

## 5. Conclusions

In conclusion, we found that RA was an important risk factor for the development of CVD. In particular, the association between CVD and RA was greater in females and younger individuals. The association between ischemic stroke and RA was significant in non-DM patients. These study findings indicate that more integrated and targeted CV prevention strategies will be important to help mitigate CVD risk in RA populations.

## Figures and Tables

**Table 1:** Baseline characteristics of the study population.

Variables	Patients with RA (*n* = 136,469)	Non-RA Matched Controls (*n* = 682,345)	*p*-Value
Age (years)	54.6 ± 11.6	54.6 ± 11.6	1
<40	12,943 (9.5)	64,715 (9.5)	
40–64	95,857 (70.2)	479,285 (70.2)	
≥65	27,669 (20.3)	138,345 (20.3)	
Sex			1
Male	36,075 (26.4)	180,375 (26.4)	
Female	100,394 (73.6)	501,970 (73.6)	
Obesity	44,023 (32.3)	224,689 (33.0)	<0.0001
Comorbidities			
Hypertension	46,501 (34.1)	218,246 (32.0)	<0.0001
Diabetes mellitus	15,100 (11.1)	73,297 (10.7)	0.0005
Hyperlipidemia	42,222 (31.0)	195,482 (28.7)	<0.0001
CKD	7171 (5.3)	34,272 (5.0)	0.0004
Low income (lowest 25%)	27,557 (20.2)	138,664 (20.3)	0.2801
BMI (kg/m^2^)	23.7 ± 3.2	23.8 ± 3.3	<0.0001
Smoking	16,352 (12.0)	84,278 (12.4)	0.0002
Alcohol drinking	41,929 (30.7)	227,156 (33.4)	<0.0001
Regular exercise	25,124 (18.4)	136,120 (20.0)	<0.0001
WC (cm)	79.4 ± 9.1	79.4 ± 9.1	0.3348
Systolic BP (mmHg)	121.5 ± 14.9	122.2 ± 15.2	<0.0001
Diastolic BP (mmHg)	75.3 ± 9.8	75.7 ± 10.0	<0.0001
Fasting glucose (mg/dL)	97.6 ± 22.1	98.9 ± 23.3	<0.0001
Total C (mg/dL)	196.8 ± 38.7	199.4 ± 37.6	<0.0001
eGFR (mL/min/1.73 m^2^)	90.7 ± 37.1	90.0 ± 37.7	<0.0001

Data are expressed as mean ± SD or *n* (%). RA, rheumatoid arthritis; CKD, chronic kidney disease; BMI, body mass index; WC, waist circumference; BP, blood pressure; C, cholesterol; eGFR, estimated glomerular filtration rate; No, number.

**Table 2:** Associations between CV events and rheumatoid arthritis.

	Subjects (*n*)	Events (*n*)	Follow-Up Duration (PYs)	Incident Rate *	Hazard Ratio (95% Confidence Interval)			
					Model 1	Model 2	Model 3	Model 4
MI								
Control	682,345	6751	3238,554.1	2.08	1 (Ref.)	1 (Ref.)	1 (Ref.)	1 (Ref.)
RA	136,469	2061	643,279.58	3.20	1.53 (1.46, 1.61)	1.55 (1.48, 1.63)	1.55 (1.48, 1.63)	1.54 (1.47, 1.62)
Stroke								
Control	682,345	7552	3233,820.4	2.33	1 (Ref.)	1 (Ref.)	1 (Ref.)	1 (Ref.)
RA	136,469	1830	643,216.59	2.84	1.23 (1.17, 1.29)	1.23 (1.17, 1.30)	1.23 (1.17, 1.30)	1.23 (1.16, 1.29)

* Incidence per 1000 person-years; Ref: reference. Model 1: non-adjusted; Model 2: adjusted for age and sex; Model 3: adjusted for variables in model 2 + smoking, drinking, regular exercise, low income, and obesity; Model 4: adjusted for variables in model 3 + hypertension, diabetes mellitus, hyperlipidemia, and chronic kidney disease. MI, myocardial infarction

## Data Availability

Data available on request from corresponding authors.

## Supplementary Materials

The following supporting information can be downloaded at: https://www.mdpi.com/article/10.3390/jcm11226812/s1, Table S1. Stratified analysis–Myocardial infarction; Table S2. Stratified analysis–Stroke; Table S3. Baseline characteristics of the study population before and after PSM; Table S4. Association between CV event and RA in the PSM cohort.

## References

[1] Avina-Zubieta J.A., Thomas J., Sadatsafavi M., Lehman A.J., Lacaille D. (2012). Risk of incident cardiovascular events in patients with rheumatoid arthritis: A meta-analysis of observational studies. Ann. Rheum. Dis..

[2] Peters M.J., van Halm V.P., Voskuyl A.E., Smulders Y.M., Boers M., Lems W.F., Visser M., Stehouwer C.D.A., Dekker J.M., Nijpels G. (2009). Does rheumatoid arthritis equal diabetes mellitus as an independent risk factor for cardiovascular disease? A prospective study. Arthritis Rheum..

[3] Van Halm V.P., Peters M.J., Voskuyl A.E., Boers M., Lems W.F., Visser M., Stehouwer C.D.A., Spijkerman A.M.W., Dekker J.M., Nijpels G. (2009). Rheumatoid arthritis versus diabetes as a risk factor for cardiovascular disease: A cross-sectional study, the CARRE Investigation. Ann. Rheum. Dis..

[4] Nikiphorou E., de Lusignan S., Mallen C.D., Khavandi K., Bedarida G., Buckley C.D., Galloway J., Raza K. (2020). Cardiovascular risk factors and outcomes in early rheumatoid arthritis: A population-based study. Heart.

[5] Chung W.S., Lin C.L., Peng C.L., Chen Y.F., Lu C.C., Sung F.C., Kao C.-H. (2013). Rheumatoid arthritis and risk of acute myocardial infarction—A nationwide retrospective cohort study. Int. J. Cardiol..

[6] Fischer L.M., Schlienger R.G., Matter C., Jick H., Meier C.R. (2004). Effect of rheumatoid arthritis or systemic lupus erythematosus on the risk of first-time acute myocardial infarction. Am. J. Cardiol..

[7] Holmqvist M., Gränsmark E., Mantel A., Alfredsson L., Jacobsson L.T., Wallberg-Jonsson S., Askling J. (2013). Occurrence and relative risk of stroke in incident and prevalent contemporary rheumatoid arthritis. Ann. Rheum. Dis..

[8] Lee T.H., Song G.G., Choi S.J., Seok H., Jung J.H. (2019). Relationship of rheumatoid arthritis and coronary artery disease in the Korean population: A nationwide cross-sectional study. Adv. Rheumatol..

[9] Lindhardsen J., Ahlehoff O., Gislason G.H., Madsen O.R., Olesen J.B., Torp-Pedersen C., Hansen P.R. (2011). The risk of myocardial infarction in rheumatoid arthritis and diabetes mellitus: A Danish nationwide cohort study. Ann. Rheum. Dis..

[10] Maradit-Kremers H., Crowson C.S., Nicola P.J., Ballman K.V., Roger V.L., Jacobsen S.J., Gabriel S.E. (2005). Increased unrecognized coronary heart disease and sudden deaths in rheumatoid arthritis: A population-based cohort study. Arthritis Rheum..

[11] Nadareishvili Z., Michaud K., Hallenbeck J.M., Wolfe F. (2008). Cardiovascular, rheumatologic, and pharmacologic predictors of stroke in patients with rheumatoid arthritis: A nested, case-control study. Arthritis Rheum..

[12] Naranjo A., Sokka T., Descalzo M.A., Calvo-Alén J., Hørslev-Petersen K., Luukkainen R.K., Combe B., Burmester G.R., Devlin J., Ferraccioli G. (2008). Cardiovascular disease in patients with rheumatoid arthritis: Results from the QUEST-RA study. Arthritis Res. Ther..

[13] Solomon D.H., Goodson N.J., Katz J.N., Weinblatt M.E., Avorn J., Setoguchi S., Canning C., Schneeweiss S. (2006). Patterns of cardiovascular risk in rheumatoid arthritis. Ann. Rheum. Dis..

[14] Solomon D.H., Karlson E.W., Rimm E.B., Cannuscio C.C., Mandl L.A., Manson J.E., Stampfer M.J., Curhan G.C. (2003). Cardiovascular morbidity and mortality in women diagnosed with rheumatoid arthritis. Circulation.

[15] Lee D.H., Sheen S.H., Lee D.G., Jang J.W., Lee D.C., Shin S.H., Han I.-b., Hong J.B., Kim H., Sohn S. (2021). Association between ischemic stroke and seropositive rheumatoid arthritis in Korea: A nationwide longitudinal cohort study. PLoS ONE.

[16] Lee J.K., Kim H., Hong J.B., Sheen S.H., Han I.B., Sohn S. (2020). Association of acute myocardial infarction with seropositive rheumatoid arthritis in Korea: A nationwide longitudinal cohort study. J. Clin. Neurosci..

[17] Mackey R.H., Kuller L.H., Deane K.D., Walitt B.T., Chang Y.F., Holers V.M., Robinson W.H., Tracy R.P., Hlatky M.A., Eaton C.B. (2015). Rheumatoid Arthritis, Anti-Cyclic Citrullinated Peptide Positivity, and Cardiovascular Disease Risk in the Women’s Health Initiative. Arthritis Rheumatol..

[18] López-Longo F.J., Oliver-Miñarro D., de la Torre I., González-Díaz de Rábago E., Sánchez-Ramón S., Rodríguez-Mahou M., Paravisini A., Monteagudo I., González C.-M., García-Castro M. (2009). Association between anti-cyclic citrullinated peptide antibodies and ischemic heart disease in patients with rheumatoid arthritis. Arthritis Rheum..

[19] Sokolove J., Brennan M.J., Sharpe O., Lahey L.J., Kao A.H., Krishnan E., Edmundowicz D., Lepus C.M., Wasko M.C., Robinson W.H. (2013). Brief report: Citrullination within the atherosclerotic plaque: A potential target for the anti-citrullinated protein antibody response in rheumatoid arthritis. Arthritis Rheum..

[20] Bacani A.K., Gabriel S.E., Crowson C.S., Heit J.A., Matteson E.L. (2012). Noncardiac vascular disease in rheumatoid arthritis: Increase in venous thromboembolic events?. Arthritis Rheum..

[21] Semb A.G., Kvien T.K., Aastveit A.H., Jungner I., Pedersen T.R., Walldius G., Holme I. (2010). Lipids, myocardial infarction and ischaemic stroke in patients with rheumatoid arthritis in the Apolipoprotein-related Mortality RISk (AMORIS) Study. Ann. Rheum. Dis..

[22] Södergren A., Stegmayr B., Ohman M.L., Wållberg-Jonsson S. (2009). Increased incidence of stroke and impaired prognosis after stroke among patients with seropositive rheumatoid arthritis. Clin. Exp. Rheumatol..

[23] Turesson C., Jarenros A., Jacobsson L. (2004). Increased incidence of cardiovascular disease in patients with rheumatoid arthritis: Results from a community based study. Ann. Rheum. Dis..

[24] Nam G.E., Kim W., Han K., Lee C.W., Kwon Y., Han B., Park S., Park J.-H., Kim Y.-H., Kim D.-H. (2020). Body Weight Variability and the Risk of Cardiovascular Outcomes and Mortality in Patients with Type 2 Diabetes: A Nationwide Cohort Study. Diabetes Care.

[25] Kim S., Kim M.S., You S.H., Jung S.Y. (2020). Conducting and Reporting a Clinical Research Using Korean Healthcare Claims Database. Korean J. Fam. Med..

[26] Kyoung D.S., Kim H.S. (2022). Understanding and Utilizing Claim Data from the Korean National Health Insurance Service (NHIS) and Health Insurance Review & Assessment (HIRA) Database for Research. J. Lipid Atheroscler..

[27] Cho S.K., Sung Y.K., Choi C.B., Kwon J.M., Lee E.K., Bae S.C. (2013). Development of an algorithm for identifying rheumatoid arthritis in the Korean National Health Insurance claims database. Rheumatol. Int..

[28] Curtis J.R., Xie F., Zhou H., Salchert D., Yun H. (2020). Use of ICD-10 diagnosis codes to identify seropositive and seronegative rheumatoid arthritis when lab results are not available. Arthritis Res. Ther..

[29] Lee S.R., Choi E.K., Jung J.H., Han K.D., Oh S., Lip G.Y.H. (2021). Lower risk of stroke after alcohol abstinence in patients with incident atrial fibrillation: A nationwide population-based cohort study. Eur. Heart J..

[30] Choi E.K. (2020). Cardiovascular Research Using the Korean National Health Information Database. Korean Circ. J..

[31] Austin P.C. (2011). An Introduction to Propensity Score Methods for Reducing the Effects of Confounding in Observational Studies. Multivar. Behav. Res..

[32] Appelman Y., van Rijn B.B., Ten Haaf M.E., Boersma E., Peters S.A. (2015). Sex differences in cardiovascular risk factors and disease prevention. Atherosclerosis.

[33] Baka Z., Buzás E., Nagy G. (2009). Rheumatoid arthritis and smoking: Putting the pieces together. Arthritis Res. Ther..

[34] Gonzalez A., Maradit Kremers H., Crowson C.S., Ballman K.V., Roger V.L., Jacobsen S.J., O’Fallon W.M., Gabriel S.E. (2008). Do cardiovascular risk factors confer the same risk for cardiovascular outcomes in rheumatoid arthritis patients as in non-rheumatoid arthritis patients?. Ann. Rheum. Dis..

[35] Liao K.P., Solomon D.H. (2013). Traditional cardiovascular risk factors, inflammation and cardiovascular risk in rheumatoid arthritis. Rheumatology.

[36] Kerola A.M., Rollefstad S., Semb A.G. (2021). Atherosclerotic Cardiovascular Disease in Rheumatoid Arthritis: Impact of Inflammation and Antirheumatic Treatment. Eur. Cardiol..

[37] Schieir O., Tosevski C., Glazier R.H., Hogg-Johnson S., Badley E.M. (2017). Incident myocardial infarction associated with major types of arthritis in the general population: A systematic review and meta-analysis. Ann. Rheum. Dis..

[38] Atzeni F., Rodríguez-Carrio J., Popa C.D., Nurmohamed M.T., Szűcs G., Szekanecz Z. (2021). Cardiovascular effects of approved drugs for rheumatoid arthritis. Nat. Rev. Rheumatol..

[39] Van den Oever I.A., van Sijl A.M., Nurmohamed M.T. (2013). Management of cardiovascular risk in patients with rheumatoid arthritis: Evidence and expert opinion. Ther. Adv. Musculoskelet. Dis..

[40] Bartoloni E., Shoenfeld Y., Gerli R. (2011). Inflammatory and autoimmune mechanisms in the induction of atherosclerotic damage in systemic rheumatic diseases: Two faces of the same coin. Arthritis Care Res..

[41] Crowson C.S., Rollefstad S., Ikdahl E., Kitas G.D., van Riel P., Gabriel S.E., Matteson E.L., Kvien T.K., Douglas K., Sandoo A. (2018). Impact of risk factors associated with cardiovascular outcomes in patients with rheumatoid arthritis. Ann. Rheum. Dis..

[42] Libby P. (2006). Inflammation and cardiovascular disease mechanisms. Am. J. Clin. Nutr..

[43] Skeoch S., Bruce I.N. (2015). Atherosclerosis in rheumatoid arthritis: Is it all about inflammation?. Nat. Rev. Rheumatol..

[44] Gonzalez-Juanatey C., Testa A., Garcia-Castelo A., Garcia-Porrua C., Llorca J., Gonzalez-Gay M.A. (2004). Active but transient improvement of endothelial function in rheumatoid arthritis patients undergoing long-term treatment with anti-tumor necrosis factor alpha antibody. Arthritis Rheum..

[45] Myasoedova E., Chandran A., Ilhan B., Major B.T., Michet C.J., Matteson E.L., Crowson C.S. (2016). The role of rheumatoid arthritis (RA) flare and cumulative burden of RA severity in the risk of cardiovascular disease. Ann. Rheum. Dis..

[46] Navarro-Millán I., Yang S., DuVall S.L., Chen L., Baddley J., Cannon G.W., Delzell E.S., Zhang J., Safford M.M., Ptkar N.M. (2016). Association of hyperlipidaemia, inflammation and serological status and coronary heart disease among patients with rheumatoid arthritis: Data from the National Veterans Health Administration. Ann. Rheum. Dis..

[47] Gonzalez-Gay M.A., Gonzalez-Juanatey C., Lopez-Diaz M.J., Piñeiro A., Garcia-Porrua C., Miranda-Filloy J.A., Ollier W.E.R., Martin J., Llorca J. (2007). HLA-DRB1 and persistent chronic inflammation contribute to cardiovascular events and cardiovascular mortality in patients with rheumatoid arthritis. Arthritis Rheum..

[48] Mantel Ä., Holmqvist M., Nyberg F., Tornling G., Frisell T., Alfredsson L., Askling J. (2015). Risk factors for the rapid increase in risk of acute coronary events in patients with new-onset rheumatoid arthritis: A nested case-control study. Arthritis Rheumatol..

[49] Solomon D.H., Reed G.W., Kremer J.M., Curtis J.R., Farkouh M.E., Harrold L.R., Hochberg M.C., Tsao P., Greenberg J.D. (2015). Disease activity in rheumatoid arthritis and the risk of cardiovascular events. Arthritis Rheumatol..

[50] Myasoedova E., Gabriel S.E., Matteson E.L., Davis J.M., Therneau T.M., Crowson C.S. (2017). Decreased Cardiovascular Mortality in Patients with Incident Rheumatoid Arthritis (RA) in Recent Years: Dawn of a New Era in Cardiovascular Disease in RA?. J. Rheumatol..

[51] Arts E.E., Fransen J., Den Broeder A.A., van Riel P., Popa C.D. (2017). Low disease activity (DAS28 ≤ 3.2) reduces the risk of first cardiovascular event in rheumatoid arthritis: A time-dependent Cox regression analysis in a large cohort study. Ann. Rheum. Dis..

[52] Fransen J., Kazemi-Bajestani S.M., Bredie S.J., Popa C.D. (2016). Rheumatoid Arthritis Disadvantages Younger Patients for Cardiovascular Diseases: A Meta-Analysis. PLoS ONE.

[53] Rohrich D.C., van de Wetering E.H.M., Rennings A.J., Arts E.E., Meek I.L., den Broeder A.A., Fransen J., Popa C.D. (2021). Younger age and female gender are determinants of underestimated cardiovascular risk in rheumatoid arthritis patients: A prospective cohort study. Arthritis Res. Ther..

[54] Cutolo M., Straub R.H. (2006). Stress as a risk factor in the pathogenesis of rheumatoid arthritis. Neuroimmunomodulation.

[55] Kerola A.M., Kerola T., Kauppi M.J., Kautiainen H., Virta L.J., Puolakka K., Nieminen T.V. (2013). Cardiovascular comorbidities antedating the diagnosis of rheumatoid arthritis. Ann. Rheum. Dis..

[56] Medeiros G., Azevedo K.P.M., Mesquita G.X.B., Lima S., Silva D.F.O., Pimenta I., Gonçalves A.K.d., Lyra C.d., Piuvezam G. (2019). Red meat consumption, risk of incidence of cardiovascular disease and cardiovascular mortality, and the dose-response effect: Protocol for a systematic review and meta-analysis of longitudinal cohort studies. Medicine.

[57] Jin J., Li J., Gan Y., Liu J., Zhao X., Chen J., Zhang R., Zhong Y., Chen X., Wu L. (2021). Red meat intake is associated with early onset of rheumatoid arthritis: A cross-sectional study. Sci. Rep..

[58] Santilli F., D’Ardes D., Guagnano M.T., Davi G. (2017). Metabolic Syndrome: Sex-Related Cardiovascular Risk and Therapeutic Approach. Curr. Med. Chem..

[59] Arts E., Fransen J., Lemmers H., Stalenhoef A., Joosten L., van Riel P., Popa C.D. (2012). High-density lipoprotein cholesterol subfractions HDL2 and HDL3 are reduced in women with rheumatoid arthritis and may augment the cardiovascular risk of women with RA: A cross-sectional study. Arthritis Res. Ther..

[60] Sammaritano L.R. (2012). Menopause in patients with autoimmune diseases. Autoimmun. Rev..

[61] Bove R. (2013). Autoimmune diseases and reproductive aging. Clin. Immunol..

[62] Pfeifer E.C., Crowson C.S., Amin S., Gabriel S.E., Matteson E.L. (2014). The influence of early menopause on cardiovascular risk in women with rheumatoid arthritis. J. Rheumatol..

