# Supplementary material for: Associations between Cardiovascular Outcomes and Rheumatoid Arthritis: A Nationwide Population-Based Cohort Study
Source: J Clin Med. 2022 Nov 17;11(22):6812. doi: 10.3390/jcm11226812 (PMC9695475; doi:10.3390/jcm11226812)
Supplement: Supplementary file 1 [file jcm-11-06812-s001.zip › jcm-2002034-supplementary.pdf]

Supplementary Table S1. Stratified analysis – Myocardial infarction.

	RA (n = 136,469)	Non-RA control (n = 682,345)			
	Incident rate (per 1,000 PYs)	Incident rate (per 1,000 PYs)	Crude HR (95% CI)	Adjusted HR* (95% CI)	*p* for interaction
**Age**					**<.0001**
**<40**	**1.07**	**0.36**	**2.99(2.2,4.07)**	**2.99(2.20,4.08)**	
**40–64**	**2.38**	**1.55**	**1.53(1.43,1.64)**	**1.52(1.42,1.63)**	
**≥65**	**7.32**	**4.86**	**1.51(1.40,1.63)**	**1.51(1.40,1.63)**	
**Sex**					**0.0293**
**Male**	**4.11**	**2.91**	**1.43(1.32,1.56)**	**1.42(1.30,1.54)**	
**Female**	**2.9**	**1.81**	**1.62(1.52,1.72)**	**1.60(1.51,1.70)**	
Low income					0.5757
No	3.18	2.05	1.56(1.47,1.65)	1.55(1.47,1.64)	
Yes	3.28	2.24	1.47(1.32,1.64)	1.53(1.41,1.65)	
Obesity					0.7111
No	2.83	1.83	1.57(1.47,1.67)	1.55(1.46,1.65)	
Yes	4.01	2.61	1.53(1.42,1.66)	1.53(1.41,1.65)	
Smoking					
No	3.05	1.94	1.57(1.49,1.66)	1.57(1.49,1.66)	0.1316
Yes	4.41	3.11	1.42(1.26,1.61)	1.39(1.23,1.57)	
Alcohol drinking					0.6955
No	3.43	2.23	1.54(1.45,1.63)	1.55(1.46,1.64)	
Yes	2.68	1.78	1.51(1.37,1.66)	1.51(1.37,1.67)	
Regular exercise					0.4476
No	3.25	2.10	1.55(1.47,1.64)	1.56(1.47,1.64)	
Yes	2.98	2.01	1.49(1.32,1.67)	1.48(1.31,1.67)	
Hypertension					0.1213
No	2.18	1.48	1.47(1.37,1.58)	1.48(1.38,1.59)	
Yes	5.21	3.36	1.55(1.45,1.66)	1.60(1.49,1.71)	
**Diabetes mellitus**					**0.0005**
**No**	**2.88**	**1.79**	**1.61(1.52,1.70)**	**1.61(1.53,1.71)**	
**Yes**	**5.99**	**4.65**	**1.29(1.16,1.44)**	**1.30(1.17,1.45)**	
Hyperlipidemia					0.1854
No	2.68	1.82	1.47(1.38,1.57)	1.50(1.41,1.60)	
Yes	4.46	2.79	1.60(1.48,1.73)	1.60(1.48,1.73)	
Chronic kidney disease					0.1018
No	3.03	1.94	1.56(1.48,1.65)	1.57(1.49,1.65)	

Yes	6.23	4.67	1.34(1.15,1.55)	1.38(1.19,1.60)

*Adjusted for age, sex, smoking, drinking, regular exercise, low income, obesity, hypertension, diabetes mellitus,
hyperlipidemia, and chronic kidney disease
In the stratification analysis, each variable used for stratification was excluded from the adjustment variables.
RA, rheumatoid arthritis; HR, hazard ratio; CI, confidence interval

Supplementary Table S2. Stratified analysis – Stroke.

	RA (n = 136,469)	Non-RA control (n = 682,345)			
	Incident rate (per 1,000 PYs)	Incident rate (per 1,000 PYs)	Crude HR (95% CI)	Adjusted HR* (95% CI)	*p* for interaction
**Age**					**0.0122**
**<40**	**0.47**	**0.20**	**2.38(1.53,3.71)**	**2.35(1.51,3.66)**	
**40–64**	**1.77**	**1.44**	**1.23(1.14,1.33)**	**1.22(1.13,1.32)**	
**≥65**	**8.01**	**6.65**	**1.21(1.12,1.29)**	**1.21(1.13,1.30)**	
**Sex**					**0.037**
**Male**	**3.70**	**3.29**	**1.12(1.03,1.23)**	**1.14(1.04,1.24)**	
**Female**	**2.60**	**2.01**	**1.27(1.20,1.35)**	**1.27(1.20,1.36)**	
Low income					0.878
No	2.78	2.27	1.23(1.16,1.30)	1.22(1.16,1.30)	
Yes	3.09	2.58	1.20(1.08,1.34)	1.22(1.10,1.37)	
Obesity					0.2887
No	2.64	2.13	1.24(1.16,1.32)	1.25(1.17,1.33)	
Yes	3.24	2.76	1.20(1.09,1.29)	1.18(1.09,1.29)	
Smoking					0.5354
No	2.74	2.23	1.24(1.18,1.31)	1.23(1.17,1.30)	
Yes	3.67	3.13	1.19(1.04,1.36)	1.11(1.04,1.35)	
Alcohol drinking					0.0673
No	3.11	2.5	1.24(1.17,1.32)	1.26(1.19,1.33)	
Yes	2.22	1.98	1.12(1.01,1.24)	1.13(1.02,1.25)	
Regular exercise					0.2083
No	2.85	2.38	1.20(1.13,1.27)	1.21(1.14,1.28)	
Yes	2.81	2.14	1.32(1.17,1.49)	1.31(1.16,1.48)	
Hypertension					0.3014

No	1.77	1.42	1.25(1.15,1.35)	1.26(1.17,1.37)	
Yes	4.96	4.3	1.15(1.08,1.23)	1.20(1.12,1.28)	
Diabetes mellitus					0.2814
No	2.44	1.97	1.24(1.17,1.31)	1.24(1.17,1.32)	
Yes	6.32	5.5	1.151(1.036,1.28)	1.16(1.05,1.29)	
Hyperlipidemia					0.648
No	2.50	2.07	1.21(1.13,1.29)	1.24(1.16,1.32)	
Yes	3.67	3.04	1.21(1.11,1.31)	1.21(1.11,1.31)	
Chronic kidney disease					0.9557
No	2.63	2.15	1.22(1.16,1.29)	1.23(1.16,1.30)	
Yes	6.61	5.62	1.18(1.02,1.36)	1.21(1.05,1.40)	

*Adjusted for age, sex, smoking, drinking, regular exercise, low income, obesity, hypertension, diabetes mellitus, hyperlipidemia, and chronic kidney disease
In the stratification analysis, each variable used for stratification was excluded from the adjustment variables.

RA, rheumatoid arthritis; HR, hazard ratio; CI, confidence interval

Supplementary Table S3. Baseline characteristics of the study population before and after PSM.

Before PSM (Table 1)					After PSM				
Variables	Patients with RA (N = 136,469)	Non-RA matched controls (N = 682,345)	P-value	ASMD*	Variables	Patients with RA (N = 136,468)	Non-RA matched controls (N = 136,468)	P-value	ASMD*
Age (years)	54.6±11.6	54.6±11.6	1	0.0000	Age (years)	54.64±11.63	54.68±11.67	0.3438	0.0036
< 40	12,943(9.5)	64,715(9.5)			< 40	12943(9.48)	12941(9.48)		
40–64	95,857(70.2)	479,285(70.2)			40–64	95856(70.24)	95703(70.13)		
≥ 65	27,669(20.3)	138,345(20.3)			≥ 65	27669(20.28)	27824(20.39)		
Sex			1	0.0000	Sex			0.9654	0.0002
Male	36,075(26.4)	180,375(26.4)			Male	36074(26.43)	36064(26.43)		
Female	100,394(73.6)	501,970(73.6)			Female	100394(73.57)	100404(73.57)		
Obesity	44,023(32.3)	224,689(33.0)	<.0001	0.0143	Obesity	44023(32.26)	44052(32.28)	0.9055	0.0005
Comorbidities					Comorbidities				
Hypertension	46,501(34.1)	218,246(32.0)	<.0001	0.0444	Hypertension	46500(34.07)	46777(34.28)	0.2636	0.0043
Diabetes mellitus	15,100(11.1)	73,297(10.7)	0.0005	0.0104	Diabetes mellitus	15099(11.06)	15234(11.16)	0.411	0.0031
Hyperlipidemia	42,222(31.0)	195,482(28.7)	<.0001	0.0501	Hyperlipidemia	42221(30.94)	42309(31)	0.7156	0.0014
CKD	7,171(5.3)	34,272(5.0)	0.0004	0.0105	CKD	7171(5.25)	7221(5.29)	0.6685	0.0016
Low income (lowest 25%)	27,557(20.2)	138,664(20.3)	0.2801	0.0032	Low income (lowest 25%)	27557(20.19)	27668(20.27)	0.5969	0.0020
BMI (kg/m2)	23.7±3.2	23.8±3.3	<.0001	0.0264	BMI (kg/m2)	23.71±3.23	23.72±3.26	0.8765	0.0006
Smoking	16,352(12.0)	84,278(12.4)	0.0002	0.0113	Smoking	16351(11.98)	16156(11.84)	0.2492	0.0044
Alcohol drinking	41,929(30.7)	227,156(33.4)	<.0001	0.0550	Alcohol drinking	41929(30.72)	41930(30.73)	0.9967	0.0000
Regular exercise	25,124(18.4)	136,120(20.0)	<.0001	0.0391	Regular exercise	25124(18.41)	25110(18.4)	0.9449	0.000

									3
WC (cm)	79.4±9.1	79.4±9.1	0.3348	0.0029	WC (cm)	79.35±9.12	79.35±9.11	0.9387	0.0003
Systolic BP (mmHg)	121.5±14.9	122.2±15.2	<.0001	0.0478	Systolic BP (mmHg)	121.48±14.91	121.49±15.21	0.9696	0.0001
Diastolic BP (mmHg)	75.3±9.8	75.7±10.0	<.0001	0.0417	Diastolic BP (mmHg)	75.33±9.79	75.32±9.97	0.9764	0.0001
Fasting glucose (mg/dL)	97.6±22.1	98.9±23.3	<.0001	0.0557	Fasting glucose (mg/dL)	97.6±22.07	97.75±21.34	0.064	0.0071
Total C (mg/dL)	196.8±38.7	199.4±37.6	<.0001	0.0675	Total C (mg/dL)	196.78±38.7	196.64±37.95	0.3212	0.0038
eGFR (mL/min/1.73m2)	90.7±37.1	90.0±37.7	<.0001	0.0177	eGFR (mL/min/1.73m2)	90.69±37.02	90.51±41.79	0.2321	0.0046

*AbsoluteStandardizedMeanDifference

Supplementary Table S4. Association between CV event and RA in the PSM cohort.

	Subjects (n)	Events (n)	Follow-up duration (PYs)	Incident rate *	Hazard ratio (95% confidence interval)
MI					
Control	136468	1357	646597.0	2.10	1(Ref.)
RA	136468	2061	643276.5	3.20	1.53 (1.43,1.64)
Stroke					
Control	136468	1505	645862.1	2.33	1(Ref.)
